# Anti-oxidant MitoQ rescue of AWB chemosensory neuron impairment in a* C. elegans* model of X-linked Adrenoleukodystrophy

**DOI:** 10.17912/micropub.biology.000346

**Published:** 2021-01-14

**Authors:** Sanjib Guha, Aurora Pujol, Esther Dalfo

**Affiliations:** 1 University of Rochester, Department of Anesthesiology & Perioperative Medicine, Rochester, NY; 2 Neurometabolic Diseases Laboratory, Bellvitge Biomedical Research Institute (IDIBELL), 08908 L'Hospitalet de Llobregat, Barcelona, Catalonia, Spain; 3 Center for Biomedical Research on Rare Diseases (CIBERER), ISCIII, Madrid, Spain.; 4 Catalan Institution of Research and Advanced Studies (ICREA), Barcelona, Catalonia, Spain.; 5 Faculty of Medicine, University of Vic-Central University of Catalonia (UVic-UCC), 08500 Vic, Spain; 6 Institut de Neurociències, Universitat Autònoma de Barcelona, 08193 Bellaterra, Spain; 7 Departament de Bioquímica i Biologia Molecular, Universitat Autònoma de Barcelona, 08193 Bellaterra, Spain

## Abstract

X-linked Adrenoleukodystrophy (X-ALD) is a neurometabolic disorder caused by a defective peroxisomal ABCD1 transporter of very long-chain fatty acids (VLCFAs). We have characterized a nematode model of X-ALD with loss of the *pmp-4* gene, the worm orthologue of *ABCD1*. These mutants recapitulated the key hallmarks of X-ALD and importantly mitochondria targeted antioxidant MitoQ prevented axonal degeneration and locomotor disability. In this study, we further demonstrated that the AWB chemosensory neuron of the *pmp-4* mutant worm is defective, both in morphology and function. Interestingly, MitoQ could rescue both the phenotypes. Collectively, our results suggest that *C. elegans’* chemosensation might provide a novel setting for exploring peroxisomal disease related disorders.​

**Figure 1. Antixiodant MitoQ rescue of AWB impairment in pmp-4 mutant worms:**
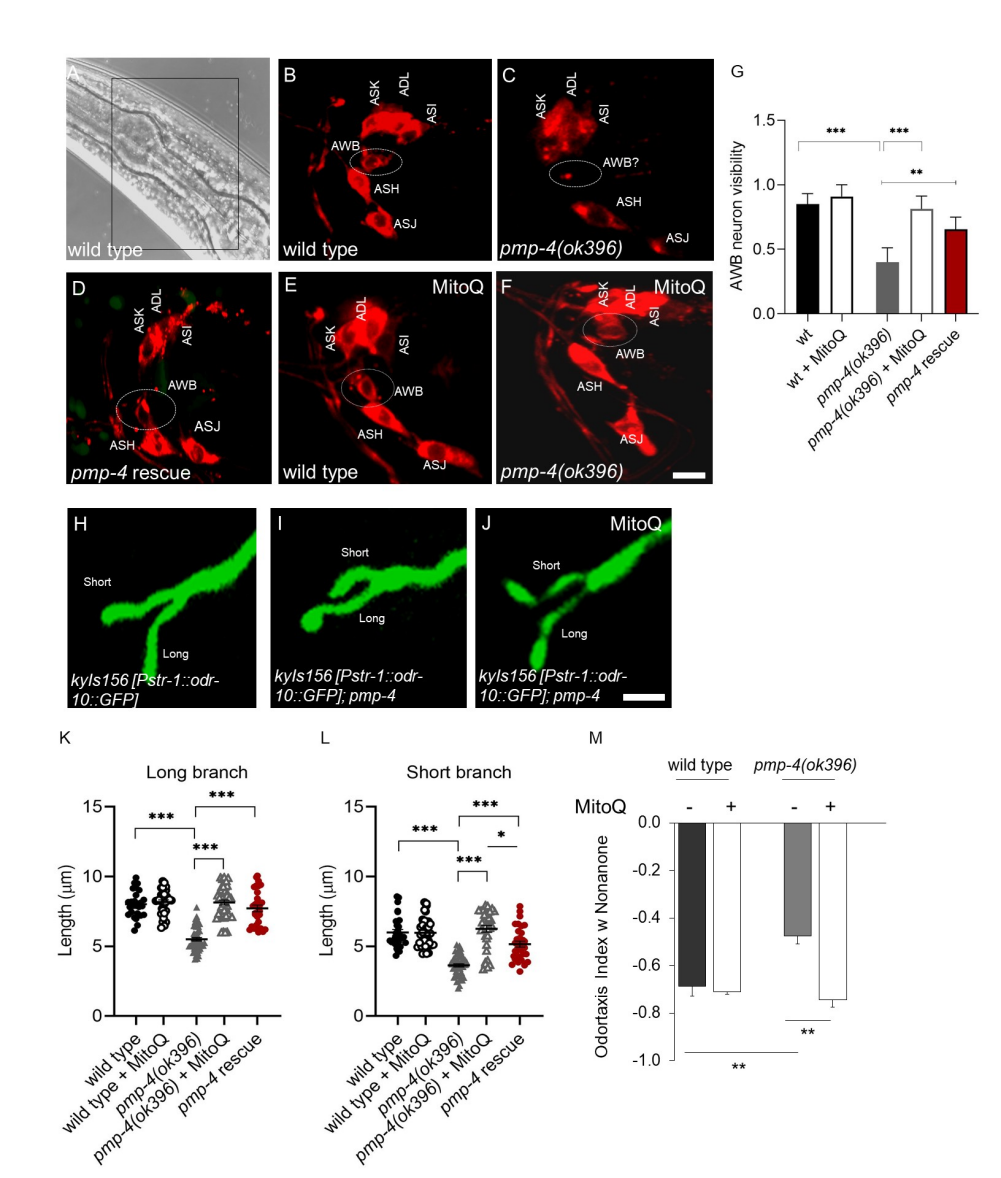
**A.** Representative DIC-Nomarski picture of an L4 worm head. B-F: DiI staining. The DiI stained neurons is delimited by the black square. **B**. Wild type animal in which all neurons stained by the DiI are visible and named. **C**. DiI staining of the *pmp-4(ok396)* mutant animal. The white dashed circle indicates limited staining of the AWB neuron **D.** DiI staining of the rescue strain EDC010 *pmp-4(ok396);ibbEx42[Ppmp-4::pmp-4::gfp].* The white dashed circle indicates the faint staining of the AWB neuron. **E, F.** Wild type and *pmp-4(ok396)* worms treated with MitoQ. Scale bar, 0.5 µm. **G.** Bar graph showing the worm fraction in which AWB neuron is visible after DiI staining, untreated and treated with MitoQ, of the corresponding genotypes (Fisher’s test *** p<0.001, **p<0.01; n = 30 animals per genotype). **H**. Confocal image (GFP channel)of the reporter strain CX3877 *kyIs156[Pstr-1::odr-10::gfp]* showing cilia of AWB neuron*.* **I, J.** Representative confocal fluorescent pictures of the AWB cilia of the *pmp-4(ok396)* worm, untreated and treated with MitoQ. Scale bar, 0.1 µm. **K, L**. Graph indicates the length of the long and short branch respectively expressed in μm, of the indicated genotypes, treated and untreated with MitoQ. N= 40 cilia analyzed per genotype. One way ANOVA, Tukey’s *post hoc* *** p<0.001, **p<0.01, *p<0.05). **M** Bar graph indicates odortaxis assay for wild type and *pmp-4* mutant worms, at baseline and treated with MitoQ. One way ANOVA, Tukey’s *post hoc* *** p<0.001, **p<0.01, *p<0.05). Each assay was conducted three times.

## Description

X-linked adrenoleukodystrophy (X-ALD) is a rare neurometabolic disease characterized by inflammatory demyelination in the brain and axonal degeneration in the spinal cord. The disease is caused by mutations in the ATP binding cassette subfamily D member (ABCD1) gene, which encodes the peroxisomal transporter of very long chain fatty acids (VLCFAs) (Ferrer *et al.*, 2010). Accumulation of VLCFAs, increased oxidative stress, and mitochondrial impairment are considered as the main etiological factor of X-ALD and other neurodegenerative disorders as reviewed in Kemp *et al.*, 2016, Turk *et al.*, 2020 and Guha *et al.*, 2020 (a).

The *C. elegans* ortholog of human ABCD1 (and ABCD2) is PMP-4, encoded by the *pmp-4* gene. We have established a novel *in vivo* model of X-ALD in *C. elegans* by using the mutant strain VC189 *pmp-4(ok396*). In this nematode model of X-ALD, PMP-4 protein was not expressed thus demonstrating that *pmp-4(ok396)* is a null allele (Coppa *et al.*, 2020*).* In our previous manuscript we have demonstrated that PMP-4 absence causes VLCFA accumulation, severe neurodegeneration and production of high amount of mitochondrial reactive oxygen species (ROS) (Coppa *et al.*, 2020). Interestingly, all these pathological phenotypes were rescued by treating worms with the mitochondrial targeted anti-oxidant drug, MitoQ (mitoquinone mesylate): which has the capability of reducing ROS generated by dysfunctional mitochondria (Coppa *et al.*, 2020).

The role of peroxisomes in lipid metabolism nowadays is more focused towards ether phospholipid biosynthesis, fatty acid alpha-oxidation and fatty acid beta-oxidation (Wanders *et al.*, 2017). In addition, peroxisomes also supply cholesterol to primary cilia, non-motile antenna-like protrusions that picks up signals required for embryonic development and adult tissue maintenance (Miyamoto *et al.*, 2020).This links peroxisomal disorders with another group of human diseases called ciliopathies, characterized by cilia dysfunction (Waters *et al.*, 2011).

Human ciliopathies have been investigated in *C. elegans* by studying the functionality of the amphid organ, the chemosensory organ located in the head of the worm, composed of glia-like cells surrounding the chemosensory neurons (Stout *et al.*, 2014; Shaham *et al.*, 2015). Considering the possibility of establishing our model as a new platform for the investigation of ciliopathies and willing to go further along this new peroxisome-cilia association (Miyamoto *et al.*, 2020), we deeply investigated the functionality of the amphid organ in the *C. elegans* model of X-ALD, constituted by the VC189 *pmp-4(ok396)* strain. In our recent study, we used two different approaches. First, the lipohilic dye 1,1′-Dioctadecyl-3,3,3′,3′-Tetramethylindocarbocyanine Perchlorate (‘DiI’; DiIC18(3), named DiI from now on, was used to visualize the dye filling behavior associated with chemosensory neurons functionality in the amphid organ (Schultz *et al.*, 2012). Second, we used reporter strain named (CX3877 *kyIs156* [*Pstr-1::odr-10::GFP*]) to visualize the structural morphology of the specific chemosensory neuron, Amphid Wing B (AWB) (Olivier Mason *et al.*, 2013). *kyIs* expresses GFP-labeled ODR-10 in STR-1 (a Seven Transmembrane Receptor involved in maintaining cell to cell connection) expressing cells (Mukhopadhyay *et al.*, 2008; Troemel *et al.*, 1997). Results obtained with the DiI stain, showed that six pairs of neurons in the head amphid sensory organs fill with DiI in wild-type animals (Fig. 1A, B). However, we found that inactivation of PMP-4 caused failure of dye uptake, specifically in the AWB neuron, but had no effect on the adjacent DiI sensitive neurons (Fig 1C). Interestingly, this dye-filling defect of *pmp-4* mutants was no longer detected by the rescue strain, containing PMP-4::GFP driven by the *pmp-4* promoter and injected in *pmp-4(ok396)* animals; *pmp-4 (ok396)* *[Ppmp-4::pmp-4(cDNA)::GFP]* ( and named rescue strain from now on for clarity) (Fig 1D) thus confirming the specificity of this dye-filling phenotype. Our previous study demonstrated that mitochondrial ROS were responsible of X-ALD associated phenotypes (Coppa *et al.*, 2020). Accordingly, we hypothesized and further investigated the *in vivo* ability of the mitochondria-targeted antioxidant, MitoQ, to protect against ROS-induced chemosensory neuronal damage in the *pmp-4* mutant animals. Worms were treated separately with the MitoQ (at 5 µg/ml) and the DiI uptake by the AWB neuron of the anti-oxidant treated worms were analyzed. Astonishingly, AWB DiI staining was rescued in all MitoQ treated *pmp-4* mutant animals (Fig 1 E, F, and G).

DiI dye-filling defect are frequently associated with defects in cilia or dendrite morphology (Olivier Mason *et al.*, 2013; Ou *et al.*, 2007) and mutants with defects in cilia or dendrite morphology frequently exhibit dye-filling defects in some or all dye-filling neurons (Olivier Mason *et al.*, 2013). To determine whether the observed dye-filing phenotype in the *pmp-4* mutant animals reflected defects in the neuronal morphology, we studied the AWB cilia structure by crossing the reporter strain *kyIs156* [*Pstr-1:: odr-10::GFP],with pmp-4(ok396)* mutant animals (Fig 1H). Generally all AWB cilia in wild type animals possess the characteristic Y-shaped structure containing two branches of different lengths (Fig 1H), the distal ends of which exhibit an irregular morphology and exhibit some animal to animal variability in the lengths of each cilial branch, depending upon which temperature they are grown and in which conditions (presence or absence of food) (Mukhopadhyay *et al.*, 2008).

However, *pmp-4* mutant animals showed both cilia branches slightly shorter than in wild-type animals (Fig. 1K, L) thus suggesting an underlying correlation between failures of DiI uptake with the regulation of cilia length (Fig. 1I, 1K, L). Interestingly, like the dye-filing defect, the AWB cilia branch length defects were recovered in the *pmp-4* rescue strain (Fig. 1K and L). Therefore PMP-4 is essential to maintain AWB cilia morphology. As we demonstrated in our previous manuscript how MitoQ rescued X-ALD associated phenotypes (Coppa *et al.*, 2020)., here also we observed that the same concentration of MitoQ also rescued the AWB distal cilia morphology in *pmp-4* mutant worms (Fig 1 J, K, L). Finally, the functional behavior associated with the AWB neuron was measured by odortaxis assay with nonanone, which generally *C. elegans* avoid (Troemel *et al.*, 1997; Tanimoto *et al.*, 2017). Odortaxis index in *pmp-4* mutant worms was decreased in comparison to wild types, and MitoQ treated animals showed a recovery in this index, as well (Fig 1N). Thus, through mitochondrial antioxidant treatment, we have demonstrated that MitoQ suppresses ROS-induced chemosensation defects in the *pmp-4 (ok396)* worms.

Apart from the X-ALD specific phenotypes described in Coppa *et al.*, 2020, in this study we have discovered that *pmp-4* mutant worms are also defective in AWB chemosensory neuron cilia structural integrity which can lead inadequate function and defective staining. This phenotype can be regulated by mitochondrial ROS, since the mitochondria specific anti-oxidant MitoQ completely rescues all the defective phenotypes. Collectively, our results suggest that *C. elegans* chemosensation phenotype can be used as a platform for the investigation of human ciliopathies in which peroxisomes are involved, and provides another setting to explore peroxisomal-related disorders.

## Methods

***C. elegans* strains growth and maintenance**

Nematodes were maintained at 20^0^C on Nematode Growth Media (NGM) plates made with Bacto Agar (BD Biosciences). The plates were seeded with live *E. coli* OP50-1 bacterial strain (cultured overnight at 37^o^C at 220 rpm) and allowed to grow overnight, as previously described in (Brenner S, 1974). For experimental assays, after synchronization by standard procedure with sodium hypochlorite, 4^th^ larval stage (L4) hermaphrodites (characterized by the appearance of a “Christmas tree vulva”) were selected and used for all the experiments.

**Dye Filling Assay**

DiI: 1,1´- Dioctadecyl-3,3,3′,3′- Tetramethylindo carbocyanine Perchlorate (Aldrich) staining was performed as previously described in (Schultz *et al.*, 2012 and Tong *et al.*, 2010). Briefly, a stock dye solution of 2 mg/ml DiI in dimethyl formamide was stored at -20ºC in a tube wrapped in foil to avoid oxidation. L4-staged well-fed worms from a plate were transferred into an eppendorf tube with 1 ml M9, into which 5 ul Dil solution from the stock was added. Tubes were shielded from light with aluminum foil and incubations were carried out at room temperature on a slow shaker for overnight. Next day, worms were mounted on a thick layer of half-dried agar (3%) pad on microscopic glass slides and subjected to confocal microscopy.

**Confocal Microscopy**

For ciliary morphology, animals were grown at the appropriate temperature were mounted on agarose pads set on microscopic slides and anaesthetized using 50 mM sodium azide in water (Sigma). Confocal images were acquired using a Leica spectral confocal microscope equipped with 63X objectives. Cilia length and morphology measurements were performed using ImageJ software (National Institutes of Health), after Z-stacking all the images, as described in Guha *et al.*, 2020(b).

**MitoQ Assay**

For rescue experiments worms were fed with MitoQ (mitoquinone mesylate): 10-(4,5-dimethoxy-2-methyl-3,6-dioxocyclohexa-1,4-dien-1-yl)decyl)triphenylphosphonium methanesulfonate, as described in (Ng *et al.*, 2014; Coppa *et al.*, 2020). One milligram of MitoQ_10_ was dissolved in 1 ml of distilled water and 250 ul (1mg/ml) was added to autoclaved and cooled to 60ºC NG agar medium (total 50_ml). Synchronized L1 worms were placed on the MitoQ and normal NGM agar plates and grown for 48 hours till they reach the L4 stage. In parallel the same experiment was performed in plates without MitoQ as a control. Finally, worms were washed of the plates with M9 buffer and similar DiL or confocal experiment was performed as mentioned before.

**Odortaxis assays**

Avoidance assays for 2-nonanone was performed on square plates as described previously (Troemel *et al.*, 1997). Plates were divided into six sectors labeled A-F. One microliter each of odorant (10% Nonanone) and 1 M NaN3 were added in two spots in sector A, and 1 μl each of control diluent (absolute ethanol) and 1 M NaN3 were added in two spots in sector F. An avoidance index (AI) or odortaxis index was calculated as [(number of animals in sectors A and B) − (number of animals in sectors E and F)]/ Total number of animals in all six sectors of the plate.

## Reagents

**Table:** 

**Genotype**	**Name**	**Source**	**Comment**
Wild type (WT)	N2	CGC	
*pmp-4(ok396) IV*	VC189	CGC	
*pmp-4(ok396) IV*	EDC1	This study	10X outcrossed
*kyIs156 [Pstr-1::odr-10::GFP]*	CX3877	CGC	GFP expression, specifically in the AWB neuron
*kyIs156 [Pstr-1::odr-10::GFP]; pmp-4*	EDC61	This study	
*ibbEx42[Ppmp-4::pmp-4::GFP::(pmp-4)3’UTR; rol-6(su1006)]*	EDC8	This study	
*pmp-4(ok396) IV; ibbEx42[Ppmp-4::pmp-4::GFP::(pmp-4)3’UTR; rol-6(su1006)]*	EDC10	This study	rescue strain used to confirm the specific role of PMP-4 in ciliary abnormalities

All the strains can be ordered on request from Prof. Dalfo’s lab which is situated in the Institut de Neurociències, Autonomous University of Barcelona, Bellaterra Campus, Catalonia, Spain.

GFP fluorescence was used to guide selection of progenies, and PCR genotyping was used to confirm homozygosity with primers specific to the *ok396 deletion*, including: 5´-TCGGTAATCCCTTGTCTTCTC-3´ (forward), 5´- CGGAGGTCATCAGGTTTGTT-3´ (reverse) and 5´- ACTCCGAAGCCGATGAAATT-3´ (deletion).

## References

[R1] Brenner S (1974). The genetics of Caenorhabditis elegans.. Genetics.

[R2] Coppa A, Guha S, Fourcade S, Parameswaran J, Ruiz M, Moser AB, Schlüter A, Murphy MP, Lizcano JM, Miranda-Vizuete A, Dalfó E, Pujol A (2020). The peroxisomal fatty acid transporter ABCD1/PMP-4 is required in the C. elegans hypodermis for axonal maintenance: A worm model for adrenoleukodystrophy.. Free Radic Biol Med.

[R3] Ferrer I, Aubourg P, Pujol A (2010). General aspects and neuropathology of X-linked adrenoleukodystrophy.. Brain Pathol.

[R4] Guha S, Johnson GVW, Nehrke K (2020). The Crosstalk Between Pathological Tau Phosphorylation and Mitochondrial Dysfunction as a Key to Understanding and Treating Alzheimer's Disease.. Mol Neurobiol.

[R5] Guha S, Fischer S, Johnson GVW, Nehrke K (2020). Tauopathy-associated tau modifications selectively impact neurodegeneration and mitophagy in a novel C. elegans single-copy transgenic model.. Mol Neurodegener.

[R6] Kemp S, Huffnagel IC, Linthorst GE, Wanders RJ, Engelen M (2016). Adrenoleukodystrophy - neuroendocrine pathogenesis and redefinition of natural history.. Nat Rev Endocrinol.

[R7] Miyamoto T, Hosoba K, Itabashi T, Iwane AH, Akutsu SN, Ochiai H, Saito Y, Yamamoto T, Matsuura S (2020). Insufficiency of ciliary cholesterol in hereditary Zellweger syndrome.. EMBO J.

[R8] Mukhopadhyay S, Lu Y, Shaham S, Sengupta P (2008). Sensory signaling-dependent remodeling of olfactory cilia architecture in C. elegans.. Dev Cell.

[R9] Ng LF, Gruber J, Cheah IK, Goo CK, Cheong WF, Shui G, Sit KP, Wenk MR, Halliwell B (2014). The mitochondria-targeted antioxidant MitoQ extends lifespan and improves healthspan of a transgenic Caenorhabditis elegans model of Alzheimer disease.. Free Radic Biol Med.

[R10] Olivier-Mason A, Wojtyniak M, Bowie RV, Nechipurenko IV, Blacque OE, Sengupta P (2013). Transmembrane protein OSTA-1 shapes sensory cilia morphology via regulation of intracellular membrane trafficking in C. elegans.. Development.

[R11] Ou G, Koga M, Blacque OE, Murayama T, Ohshima Y, Schafer JC, Li C, Yoder BK, Leroux MR, Scholey JM (2007). Sensory ciliogenesis in Caenorhabditis elegans: assignment of IFT components into distinct modules based on transport and phenotypic profiles.. Mol Biol Cell.

[R12] Schultz RD, Gumienny TL (2012). Visualization of Caenorhabditis elegans cuticular structures using the lipophilic vital dye DiI.. J Vis Exp.

[R13] Shaham S (2015). Glial development and function in the nervous system of Caenorhabditis elegans.. Cold Spring Harb Perspect Biol.

[R14] Stout RF Jr, Verkhratsky A, Parpura V (2014). Caenorhabditis elegans glia modulate neuronal activity and behavior.. Front Cell Neurosci.

[R15] Tanimoto Y, Yamazoe-Umemoto A, Fujita K, Kawazoe Y, Miyanishi Y, Yamazaki SJ, Fei X, Busch KE, Gengyo-Ando K, Nakai J, Iino Y, Iwasaki Y, Hashimoto K, Kimura KD (2017). Calcium dynamics regulating the timing of decision-making in *C. elegans*.. Elife.

[R16] Tong YG, Bürglin TR (2010). Conditions for dye-filling of sensory neurons in Caenorhabditis elegans.. J Neurosci Methods.

[R17] Troemel ER, Kimmel BE, Bargmann CI (1997). Reprogramming chemotaxis responses: sensory neurons define olfactory preferences in C. elegans.. Cell.

[R18] Turk BR, Theda C, Fatemi A, Moser AB (2020). X-linked adrenoleukodystrophy: Pathology, pathophysiology, diagnostic testing, newborn screening and therapies.. Int J Dev Neurosci.

[R19] Wanders RJ, Poll-The BT (2015). "Role of peroxisomes in human lipid metabolism and its importance for neurological development".. Neurosci Lett.

[R20] Waters AM, Beales PL (2011). Ciliopathies: an expanding disease spectrum.. Pediatr Nephrol.

